# *NCR3* polymorphism, haematological parameters, and severe malaria in Senegalese patients

**DOI:** 10.7717/peerj.6048

**Published:** 2018-12-03

**Authors:** Alassane Thiam, Sabrina Baaklini, Babacar Mbengue, Samia Nisar, Maryam Diarra, Sandrine Marquet, Mouhamadou Mansour Fall, Michel Sanka, Fatou Thiam, Rokhaya Ndiaye Diallo, Magali Torres, Alioune Dieye, Pascal Rihet

**Affiliations:** 1 Unité d’Immunogénétique, Institut Pasteur de Dakar, Dakar, Senegal; 2 Aix Marseille Univ, INSERM, TAGC, Marseille, France; 3 Service d’Immunologie, University Cheikh Anta Diop of Dakar, Dakar, Senegal; 4 G4 Biostatistique, Institut Pasteur de Dakar, Dakar, Sénégal; 5 Service de Réanimation, Hôpital Principal de Dakar, Dakar, Senegal

**Keywords:** Genetic association, Mild malaria, Severe malaria, *Plasmodium falciparum*, Host factors

## Abstract

**Background:**

Host factors, including host genetic variation, have been shown to influence the outcome of *Plasmodium falciparum* infection. Genome-wide linkage studies have mapped mild malaria resistance genes on chromosome 6p21, whereas *NCR3-412* polymorphism (rs2736191) lying within this region was found to be associated with mild malaria.

**Methods:**

Blood samples were taken from 188 *Plasmodium falciparum* malaria patients (76 mild malaria patients, 85 cerebral malaria patients, and 27 severe non-cerebral malaria patients). *NCR3-412* (rs2736191) was analysed by sequencing, and haematological parameters were measured. Finally, their association with clinical phenotypes was assessed.

**Results:**

We evidenced an association of thrombocytopenia with both cerebral malaria and severe non-cerebral malaria, and of an association of high leukocyte count with cerebral malaria. Additionally, we found no association of *NCR3-412* with either cerebral malaria, severe non-cerebral malaria, or severe malaria after grouping cerebral malaria and severe non-cerebral malaria patients.

**Conclusions:**

Our results suggest that *NCR3* genetic variation has no effect, or only a small effect on the occurrence of severe malaria, although it has been strongly associated with mild malaria. We discuss the biological meaning of these results. Besides, we confirmed the association of thrombocytopenia and high leukocyte count with severe malaria phenotypes.

## Background

*Plasmodium falciparum* malaria remains a major public health concern causing 212 million malaria cases occurred in 2015 (WHO, 2016). The most severe forms of malaria caused by *P. falciparum* are cerebral malaria, severe anaemia, and respiratory distress (Marsh & Snow, 1999). According to the WHO report, the major complications account for 429,000 deaths annually, essentially in children, pregnant woman and immuno-suppressed individuals. It should be noticed, however, that approximately 2% of the clinical cases are severe (Greenwood, 1997), while most of malarial infection cases remain asymptomatic.

Host genetic factors have been shown to influence the outcome of infection (Hernandez-Valladares, Rihet & Iraqi, 2014; Marquet, 2017). Genome scans have identified several human loci linked or associated with parasitaemia, mild malaria (Brisebarre et al., 2014; Milet et al., 2010; Sakuntabhai et al., 2008), or severe malaria (Band et al., 2013; Jallow et al., 2009; Malaria Genomic Epidemiology et al., 2015; Timmann et al., 2012). Genome scans that have been performed in mice have also identified some loci, with some corresponding to those genetically linked to parasitemia (Char 3 and Char 8) or cerebral malaria (Cmsc) in humans (Hernandez-Valladares, Rihet & Iraqi, 2014). In addition, many candidate gene studies provided evidence of an association between human genetic variants and malaria phenotypes (Driss et al., 2011; Hernandez-Valladares, Rihet & Iraqi, 2014; Kwiatkowski, 2005; Marquet, 2017). Some genes have been found to be associated with both mild and severe forms of clinical malaria. These include *HBB*, *TNF* and *NOS2A* (Kwiatkowski, 2005; Marquet, 2017). Interestingly, *TNF* is located within chromosome 6p21.3 linked to parasitemia and mild malaria in humans, and the corresponding chromosomal region in mice is genetically linked to mild malaria and cerebral malaria (Burt et al., 1999; Hernandez-Valladares et al., 2004; Ohno & Nishimura, 2004). Moreover, other genetic variants within chromosome 6p21.3 have been associated with severe malaria (Diakite et al., 2009).

*NCR3* that is also located within chromosome 6p21.3 has been shown to be associated with mild malaria in Burkina Faso (Delahaye et al., 2007) and in the Republic of Congo (Baaklini et al., 2017). Both population-based and family-based studies showed that *NCR3-412C* carriers had more frequent mild malaria attacks than *NCR3-412GG* individuals. In addition, Baaklini et al. (2017) showed that *NCR3-412* (rs2736191) alters both the binding of transcription factors to the DNA and the promoter activity. Moreover, Mavoungou et al. (2007) reported that NKp30 encoded by *NCR3* is involved in the recognition of *P. falciparum-* infected red blood cells by natural killer (NK) cells. It might be possible that *NCR3-412* alters the transcriptional level of NCR3 resulting in an effect on the amount of NKp30 at the NK cell surface and their response to infected red blood cells. To our knowledge, there was, however, no NCR3 association study with severe malaria.

Here we considered *NCR3* as a candidate gene for severe malaria, and analysed its polymorphism in *P. falciparum*-infected individuals. We genotyped *NCR3-412* (rs2736191) in DNA samples from patients including 76 mild malaria, 85 cerebral malaria, and 27 severe non-cerebral malaria subjects. We report the analysis of *NCR3-412* (rs2736191) and several haematological parameters association with severe malaria phenotypes.

## Methods

### Patients and phenotypes

The study was performed between 2012 and 2015 in two Senegalese hospitals including one in Dakar and the other in Tambacounda at the Southeast part of the country. Two categories of patients with clinical malaria namely, severe malaria (SM) and mild malaria (MM) patients were enrolled. All the clinical cases were defined according to the WHO criteria (WHO, 2000). For all patients, the presence of *P. falciparum* infection was determined by an immunoassay enabling the detection of pfHRP2 (Standard diagnostics-Abbott Inc, Chicago, Il, USA). *P. falciparum* parasitemia was assessed by microscopic examination of thin and thick smears, prior anti-malarial treatment. Clinical histories, data and basic demographic information, ethnic group, and study questionnaires were recorded at baseline for each subject. In particular, the affiliation to an ethnic group was provided by the patients or their parents. At the day of admission, biological data including parasite density, haematology and biochemistry parameters were determined at the hospitals’ medical laboratories and recorded. Briefly, thick and thin blood films were stained with 10% Giemsa solution for 15 min to measure parasitaemia. Parasite determination and measurement were established by two independent readings. The parasitaemia was defined as the number of parasitized erythrocytes observed per µl of blood. *P. falciparum* was the only *Plasmodium* species detected. Samples were taken at the admission before any treatment. An informed consent was obtained from each participant and/or their relatives prior to inclusion, after giving them written or verbal information in their native language. The protocols were approved by the investigator’s institutions, the National Ethical Committee and the Ministry of Health of Senegal.

For severe malaria, two groups of patients were defined according to the neurological dysfunction, namely cerebral malaria cases (CM) and non CM (NCM) cases. CM cases were defined on the basis of a deep coma, an unpurposeful response or no response to a painful stimulus by Glasgow score <9, a microscopically diagnosed *P. falciparum* infection, without other clinically cause of impaired consciousness, such as hypoglycemia, meningitis, and encephalitis according to WHO criteria (Saissy, Rouvin & Koulmann, 2003). NCM cases were defined as severe malaria (SM) without neurological symptoms such as impaired consciousness, convulsions and long-term neurological deficits. NCM patients presented isolated symptoms of SM such as severe anaemia, hypoglycaemia, respiratory distress, or hypoxia. The NCM group consisted of 27 patients, whereas the CM group consisted of 85 patients. The patients recruited in each hospital were managed by the medical staff. The treatment protocol was based on the Senegalese national recommendations, which are intramuscular quinine 20 mg/kg followed by 20 mg/kg every 8 h. Patients were examined every 4 h for the first 24 h and every 6 h thereafter.

Regarding MM, a total of 76 patients who were treated at the outpatient clinic of the hospital were initially enrolled. Of these, 76 patients had fever with *P. falciparum* parasitaemia of <25,000 parasites/µL of blood, with no evidence of impaired consciousness or seizures before and at the time of enrolment. MM patients were followed up for two weeks after their enrolment, and there was no case of impaired consciousness or seizures. Blood samples from MM patients were obtained on the day of hospital admission.

### DNA extraction, genome amplification, and genotyping

Blood Mini Kit (Qiagen, Hilden, Germany) was used for DNA extraction according to the manufacturer’s instructions (Koukouikila-Koussounda et al., 2013). The total DNA samples were then amplified by IllustraGenomiPhi V2 DNA Amplification kit (GE Healthcare Life Sciences, Velizy-Villacoublay, France) using the following protocol: 1 µl containing approximately 10 ng of genomic DNA was mixed with 9 µl of Sample Buffer. The mixture was heated to 95°C for 3 min to denature genomic DNA and then cooled down to 4 °C or incubated on ice. For DNA amplification, a master mix solution containing 9 µl of Reaction Buffer and 1 µl of Phi29 DNA polymerase enzyme mix was added directly in the sample. Sample were then incubated at 30 °C for 1.5 h and heated to 65 °C for 10 min to inactivate the enzyme. The amplification product was diluted up to 1 ml in total volume with pure water to reach a DNA concentration of about 4–7 ng/µl and was stored at −20 °C until used.

The primer pair to amplify DNA fragments containing *NCR3-412* (rs2736191) was designed as described (Baaklini et al., 2017); PCR amplification of DNA fragments was performed with forward (5′-GATGGGTCTGGGTACTGGTG-3′) and reverse (5′-GGGATCTGAGCAGTGAGGTC-3′) primers, respectively. For Hemoglobin S (rs334) and hemoglobin C (rs33930165), the forward primer was 5′-CTGAGGGTTTGAAGTCCAAC- 3′, and the reverse one was 5′-CAGCATCAGGAGTGGACAG-3′. The PCR products were submitted to electrophoresis on 1.5% agarose gel electrophoresis to verify the product size and were purified using a PCR DNA Purification Kit (QIAGEN). The purified PCR products were sequenced using the Sanger method (GATC Biotech, Konstanz, Germany).

### Statistical analyses

Hardy-Weinberg equilibrium was tested and allelic frequencies were calculated, as described (Rodriguez, Gaunt & Day, 2009). Other statistical tests were performed by using R or the SPSS software (SPSS, Boulogne, France). To assess the association of quantitative biological phenotypes and clinical phenotypes, we used the Kruskal-Wallis test and the Mann-whitney test. To estimate the association of rs2736191 polymorphism with malaria phenotypes, we conducted a chi-square test and a logistic regression analysis, and we evaluated the odds ratios (OR) and their 95% confidence intervals (CIs). MM group was considered the control group, whereas the case group consisted of CM patients, NCM patients, or severe malaria patients after grouping CM and NCM patients. The distribution of MM patient genotypes was compared with the one of CM, NCM, or severe malaria patient genotypes. All the tests were two-sided. G*power software was used to compute statistical power for the genetic association study.

## Results

### Association between biological parameters and severe malaria

Table 1 shows the characteristics of the patients. There was no effect of gender (Chi-square = 2.4, *df* = 2, *P* = 0.3). CM patients had a significantly higher risk of dying compared to NCM (*P* = 0.04) or MM patients (*P* = 2.0 10^−6^) by using a Fisher exact test. Moreover, there were significant differences between CM, NCM, and MM patients on the basis of the Kruskal-Wallis test for age (*P* = 0.009), erythrocyte count (*P* = 0.019), haematocrit (*P* = 3.3 10^−6^), haemoglobin concentration (*P* = 2.4 10^−6^), leucocyte count (*P* = 0.006), and platelet count (*P* = 7.9 10^−4^).

**Table 1:** Patient characteristics. Characteristics include age, gender, ethnicity, haematological parameters, and beta-globin genetic variation.

	**Cerebral malaria** (*N* = 85)	**Non cerebral malaria** (*N* = 27)	**Mild malaria** (*N* = 76)
**Gender**			
Female	28	10	34
Male	57	17	42
**Survival**			
Dead	19	1	0
Alive	66	26	76
**Ethnic group**^a^			
Bambara	8	3	5
Diola	5	3	2
Peulh	22	14	30
Serrere	8	0	9
Soninke	3	1	8
Wolof	28	4	20
Others	11	2	2
**Age**^b^			
Median	22	8	27
(25th and 75th percentile)	(12–41)	(3–33)	(13–41)
**Haematological data**^b^			
*-Red blood cells* (×10^6^/µL)			
*N*	68	23	66
Median	3.8	2.8	4.0
(25th and 75th percentile)	(3.0–4.3)	(2.4–4.1)	(3.5–4.6)
*-Haematocrit (%)*			
*N*	67	23	66
Median	30.6	20.0	35.0
(25th and 75th percentile)	(25.8–34.4)	(16.9–27.5)	(29.7–38.7)
*-Haemoglobin* (g/dL)			
*N*	80	26	66
Median	10.4	7.1	11.1
(25th and 75th percentile)	(8.4–11.9)	(5.3–9.4)	(9.8–12.5)
*-Leucocytes* (×10^3^/µL)			
*N*	77	24	66
Median (25th and 75th percentile)	11.0 (6.5–14.0)	7.9 (5.4–12.9)	7.9 (5.0–10.6)
*-Platelets* (×10^3^/µL)			
*N*	66	26	66
Median	87.5	100.5	183.0
(25th and 75th percentile)	(40.0–168.3)	(58.5–165.0)	(83.0–237.8)
*-Parasitemia* (pRBC^c^/µL)			
*N*	37	16	51
Median	560	2,800	6,545
(25th and 75th percentile)	(115–5,983)	(592–6,716)	(620–57,840)
**HBB genetic variation**^d^			
*-Hb S carrier, n (%)*	3 (3.6)	1 (3.7)	5 (6.7)
*-Hb C carrier, n (%)*	0 (0.0)	2 (7.4)	1 (1.3)

**Notes.**

a Ethnic groups are shown. Other ethnic groups that are Bassari, Cap-vert, Mandinge, and Soce groups were rare (*n* ≤ 5). The ethnic group was unknown for two individuals.

b Median and (25th and 75th percentile) are shown.

c Parasitized red blood cells.

d Two individuals have not been genotyped.

**Figure 1:**
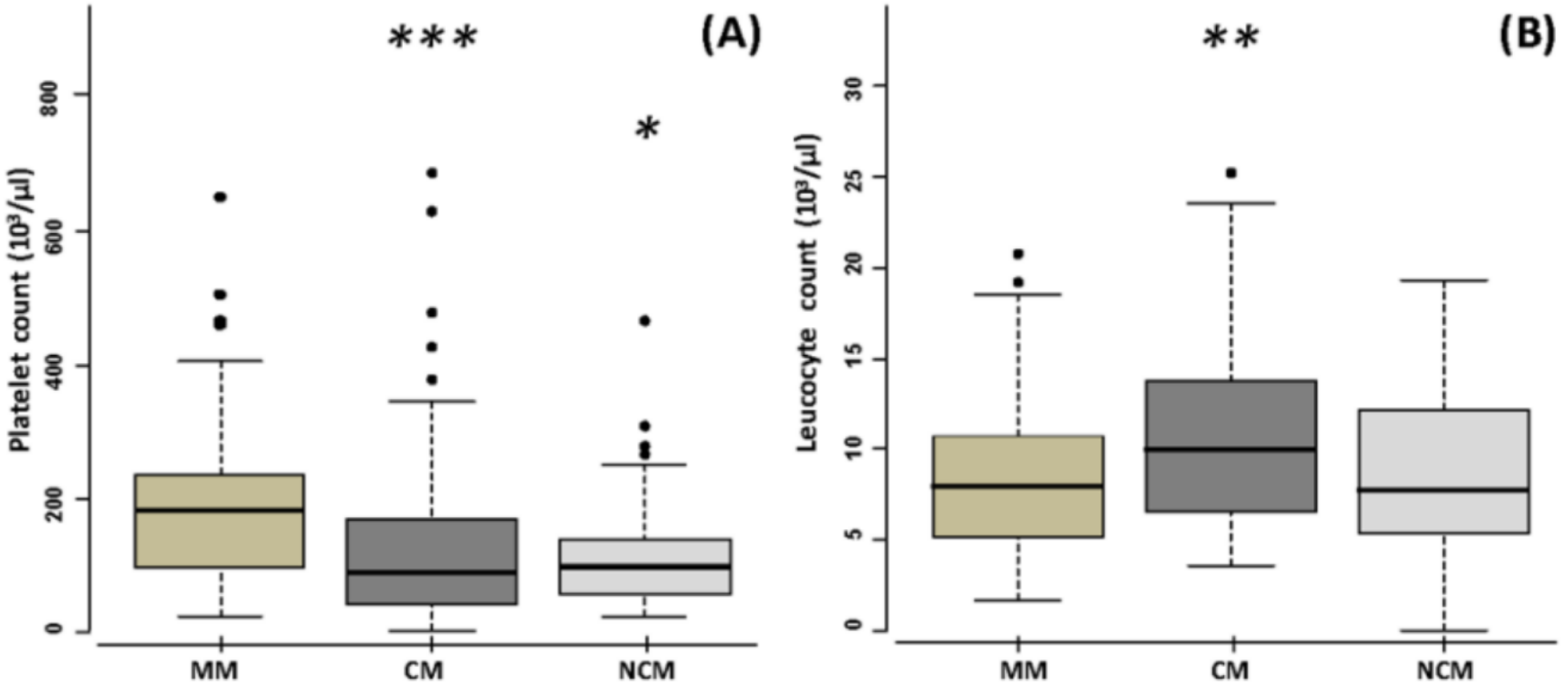
Haematological characteristics of mild malaria (MM), cerebral malaria (CM), and noncerebral malaria (NCM) patients. (A) Platelet count (×10^3^/µL). (B) Leucocyte count (×10^3^/µL). Median, 25th and 75th percentile are shown for each group. Significant differences between MM and CM, and between MM and NCM are shown. *P* values have been calculated by using Mann–Whitney test (***P < 0.001; **P < 0.01; *P < 0.05).

NCM patients were younger than CM and MM patients (*P* < 0.006) by using the Mann–Whitney method, whereas there was no difference between CM and MM for age. Erythrocyte count (*P* = 0.01), haematocrit (*P* = 4.9 10^−6^), and haemoglobin concentration (*P* = 1.4 10^−6^) of NCM patients were lower than those of MM patients. NCM patients showed a lower haematocrit (*P* = 1.1 10^−4^) and a lower haemoglobin concentration (*P* = 3.4 10^−5^) compared to CM patients. Moreover, we detected a reduction of platelet count (*P* = 0.0003) and an increase of leucocyte count (*P* = 0.0015) in CM patients compared to the ones of MM patients, as shown in Fig. 1. There was no significant difference between CM and NCM for leucocyte count (*P* = 0.14) and for platelet count (*P* = 0.64)*.* Furthermore, we found a reduction of platelet count in NCM patients compared to the ones of MM patients (*P* = 0.018), whereas there was no difference for the leucocyte count (*P* = 0.44) between these two groups.

### Influence of ethnic groups and genetic factors on severe malaria

Table 1 shows the distribution of the ethnic groups according to the clinical phenotypes. Peul and Wolof groups were the most represented ethnic groups among the patients. These groups differed in the distribution of their clinical phenotype (Chi-square = 6.4, *df* = 2, *P* = 0.04), whereas there was a trend when comparing Peul to all other ethnic groups (Chi-square = 5.6, *df* = 2, *P* = 0.06). More specifically, there was a trend in favour of a better protection from CM (OR = 0.576, 95% confidence interval 0.296–1.120), although not significant (*P* > 0.05), on the basis of the comparison of MM with CM patients. In the same way, there was a trend in favour of a lower parasitaemia mean in Peul patients, although not significant (*P* > 0.05); there was, nevertheless, a lower parasitaemia variance in Peul patients (*P* = 0.003). Haemoglobin S and haemoglobin C carriers were rare in CM, NCM, and MM patients, as shown in Table 1. There was no significant association between haemoglobin genotypes and clinical phenotypes.

Table 2 shows the distribution of *NCR3* genotype by clinical status. There were 123 GG, 52 GC, and 8 CC individuals. *NCR3-412* was in Hardy-Weinberg equilibrium (*P* = 0.77). *NCR3-412G* allele frequency was 0.81, whereas *NCR3-412C* allele frequency was 0.19. We clustered GC and CC individuals for further analyses.

**Table 2:** Clinical status of patients for *NCR3-412* polymorphism.

Genotype	Cerebral malaria	Non cerebral malaria	Mild malaria	Total
CC	5	0	3	8
GC	19	9	24	52
GG	58	18	47	123
Total	82^a^	27	74^a^	183

**Notes.**

a Three CM and 2 MM individuals have not been genotyped.

We tested the association of the *NCR3-412C* allele carriage with severe malaria by using the Chi-square test. To this aim, we compared the distribution of MM patient genotypes with the one of severe malaria patient genotypes. There was no association with CM (Chi-square = 0.9, *df* = 1, *P* = 0.34), NCM (Chi-square = 0.09, *df* = 1, *P* = 0.77), or severe malaria after grouping CM and NCM patients (Chi-square = 0.77, *df* = 1, *P* = 0.38).

We applied a logistic regression method when taking into account age and gender, which may influence malaria phenotypes. There was no association with CM (*P* = 0.56), NCM (*P* = 0.92), or severe malaria (*P* = 0.61). The odds of CM, NCM, and severe malaria between *NCR3-412GG* and *NCR3-412GG/GC* were 0.813 (95% confidence interval 0.406–1.628), 1.047 (95% confidence interval 0.396–2.769), and 0.847 (95% confidence interval 0.445–1.614), respectively. Similar results were obtained after adding haemoglobin genotypes and ethnic groups as covariates. In addition, we assessed the power of our analyses on the basis of the sample size used, an α-risk of 5%, and an effect size equal to the one detected by Delahaye et al. (2007) for the association of the *NCR3-412C* allele carriage with mild malaria. We obtained a power of 85% and 90% for CM and severe malaria, respectively.

## Discussion

A few genes have been associated with both mild malaria and severe malaria. We investigated a *NCR3* genetic variant, which was linked and associated with mild malaria in Burkina Faso (Delahaye et al., 2007). We recruited a new study population in Senegal, recorded biological parameters and ethnic groups, genotyped DNA samples for *NCR3-412*, haemoglobin C and haemoglobin S polymorphism in patients with either mild or severe malaria, and performed an association study based on a case-control design.

Reduced red blood cell count, haematocrit, and haemoglobin concentration were associated with noncerebral malaria, indicating a marked anaemia in these patients compared to mild malaria and cerebral malaria. Thrompocytopenia was associated with both cerebral malaria and severe non-cerebral malaria, as previously reported (Gerardin et al., 2002; Lampah et al., 2015; Muwonge et al., 2013). Thrompocytopenia is thought to be due to platelet sequestration and platelet consumption (Akinosoglou, Solomou & Gogos, 2012; Hochman et al., 2015; Karanikas et al., 2004). In the same way, platelet adhesion to brain endothelial cells has been associated with cerebral malaria (Grau et al., 2003). Furthermore, it has been shown to promote adhesion of infected red blood cells to TNF-stimulated endothelium (Wassmer et al., 2004), and to alter gene expression *in vitro* in human brain microvascular endothelial cells (Barbier et al., 2011), thus contributing to brain endothelial dysfunction.

Moreover, an increase of leukocyte count was associated with CM, whereas MM patients and NCM patients did not differ in their leukocyte count. In the same way, leukocyte counts were reported to be higher in severe malaria patients compared to those of mild malaria patients (Berens-Riha et al., 2014; Cserti-Gazdewich et al., 2013; Modiano et al., 2001c; Van Wolfswinkel et al., 2013), and in malaria patients with high parasitaemia compared to those of individuals with low parasitaemia (Kotepui et al., 2015; McKenzie et al., 2005). The increase of leucocyte number is consistent with a strong host inflammatory response associated with severe malaria. It may be due to polymorphonuclear leukocyte or monocyte increase (Hochman et al., 2015). T, B, and NK lymphocyte counts have been found to be reduced in severe malaria patients (Mandala et al., 2015), whereas the percentage of activated T CD4+, NK, and γδT lymphocytes were higher in cerebral malaria patients compared to mild malaria patients (Mandala et al., 2015). This is consistent with the role of T cell dependent immunopathology in the pathogenesis of cerebral malaria. This also suggests a role of NK and γδT lymphocytes in malaria immunopathology. In the same way, NK and γδT lymphocytes produce IFNγ and TNFα in malaria patients (D’Ombrain et al., 2007; Horowitz et al., 2010; Stanisic et al., 2014), while polymorphisms within the natural killer cell complex modulate mouse cerebral malaria (Hansen et al., 2014). In humans, the effect of NK polymorphisms on severe malaria has been poorly investigated, and yielded conflicting results (Olaniyan et al., 2014; Yindom et al., 2012).

Since the Peul ethnic group which has a particular genetic background has a lower risk of mild malaria attacks, a lower parasitaemia, and a higher anti-plasmodial antibody response compared to other sympatric ethnic groups (Arama et al., 2015; Modiano et al., 2001a; Modiano et al., 1996), we hypothesized that Peul patients may also show a better protection against severe malaria. Although we detected a trend in favour of this hypothesis, the association was not significant at the 5% level. This result urges, nevertheless, new studies with a bigger sample size to increase statistical power.

**Figure 2:**
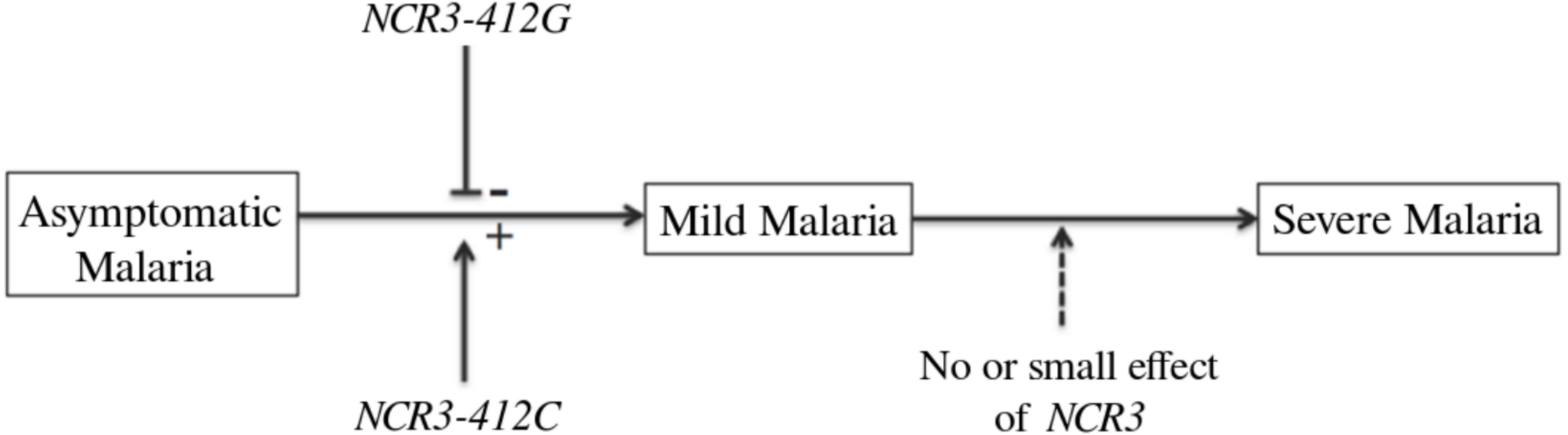
*NCR3* and severity of the disease. *NCR3-412C* allele that increased the risk of mild malaria was shown to decrease both the binding of transcription factors to the promoter and *NCR3* expression. Here we report that there was no difference between *NCR3-412* allele frequency between mild malaria patients and severe malaria patients. Therefore, *NCR3-412* polymorphism unlikely explains why some patients with mild malaria develop severe forms of the disease.

Since, haemoglobin S and haemoglobin C are well known to protect against severe malaria (Jallow et al., 2009; Modiano et al., 2001b; Timmann et al., 2012), we searched for haemoglobin S and haemoglobin C in our study population. Although we detected both mutations, the frequency of haemoglobin S and haemoglobin C was very low, and we could not provide evidence for a protective effect against severe malaria.

We further assessed the association of *NCR3-412* with severe malaria. Mild malaria patients and severe malaria patients did not differ in their *NCR3-412* allelic frequency. Power studies revealed a power of approximately 85% based on an effect size corresponding to the one found in the mild malaria studies (Delahaye et al., 2007). This indicates that the effect size is significantly lower for severe malaria or that there is no association between the SNP and severe malaria. In the former case, increasing the sample size should allow to detect an association with a small effect size. Alternatively, integrating the polymorphisms of other NK cell receptors should increase the power for detecting a putative association of NK receptors with severe malaria. In the later case, the NCR3 expression may not influence the occurrence of severe malaria. It should be stressed that very few genes have been associated both with mild and severe malaria.

If we assume that there is no association between *NCR3-412* and severe malaria, we should keep in mind that there is an association between *NCR3-412* and mild malaria (Baaklini et al., 2017; Delahaye et al., 2007). This means that there is no allele frequency difference between mild malaria patients and severe malaria patients, on the one hand, and that there is an allele frequency difference between mild malaria patients and individuals with an asymptomatic infection, on the other hand. Also, *NCR3-412* polymorphism may partly explain why individuals with asymptomatic malaria have mild malaria attacks, but is unlikely to explain why some patients with mild malaria develop severe forms of the disease (Fig. 2).

It is important to point out the limitation of our study. First, the time of our clinical investigation was limited. Also, the absence of CM or NCM was registered on the basis of the declaration of the patients or their parents. Second, it cannot be excluded that clinical status could be due to differential anti-malarial immunity response resulting from different exposure levels. Finally, the sample size of our study population is limited. In spite of this limitation, it should be stressed that we detected an association of leucocyte and platelet counts with clinical status, confirming previous results. This shows that the sample size was sufficient to allow us to detect association with a high effect size. In this way, our power analysis for genetic association indicated that we could detect an association of NCR3 with severe malaria when assuming an effect size similar to the one we calculated for mild malaria in Burkina Faso or in the Republic of Congo (Baaklini et al., 2017; Delahaye et al., 2007). Nevertheless, we cannot exclude that there was an association of NCR3 with severe malaria with a small effect size.

## Conclusions

To summarize, we conducted an epidemiological study that allowed us to confirm the association of cerebral malaria with thrombocytopenia and leukocyte count. Besides, we did not detect an association of severe malaria with *NCR3* genetic variation. This suggests that genetic variation at this locus does not influence the molecular mechanisms involved in the most severe forms of the disease, although it influences the risk of developing mild malaria attacks.

##  Supplemental Information

10.7717/peerj.6048/supp-1 Data S1 Raw data for association analyses Gender, age, ethnic group, clinical malaria phenotype, survival, NCR3 and HBB genotypes are stated for each individual.

## Abbreviations

CI: confidence interval
CM: cerebral malaria
NCM: non-cerebral malaria
MM: mild malaria
NK: natural killer
OR: Odds ratio

## Additional Information and Declarations

### Data Availability

The following information was supplied regarding data availability: The raw data are provided in Supplemental File.
